# Effects of Exendine-4 on The Differentiation of Insulin Producing Cells from Rat Adipose-Derived Mesenchymal Stem Cells

**DOI:** 10.22074/cellj.2016.3844

**Published:** 2016-01-17

**Authors:** Layasadat Khorsandi, Sadegh Saremy, Ali Khodadadi, Fereshteh Dehbashi

**Affiliations:** 1 Cell and Molecular Research Center, Faculty of Medicine, Ahvaz Jundishapur University of Medical Sciences, Ahvaz, Iran; 2 Department of Anatomical Sciences, Ahvaz Jundishapur University of Medical Sciences, Ahvaz, Iran

**Keywords:** Exendin-4, Differentiation, Insulin-Secreting Cells, Adipose Tissue, Mes- enchymal Stem Cells

## Abstract

**Objective:**

To evaluate the effect of Exendine-4 (EX-4), a Glucagon-like peptide 1 (GLP-1) receptor agonist, on the differentiation of insulin-secreting cells (IPCs) from rat adipose-derived mesenchymal stem cells(ADMSCs).

**Materials and Methods:**

In this experimental study, ADMSCs were isolated from rat adi- pose tissue and exposed to induction media with or without EX-4. After induction, the existence of IPCs was confirmed by morphology analysis, expression pattern analysis of islet-specific genes (*Pdx-1, Glut-2* and *Insulin*) and insulin synthesis and secretion.

**Results:**

IPCs induced in presence of EX-4 were morphologically similar to pancre- atic islet-like cells. Expression of *Pdx-1, Glut-2* and *Insulin* genes in EX-4 treated cells was significantly higher than the cells exposed to differentiation media without EX-4. Compared to EX-4 untreated ADMSCs, insulin release from EX-4 treated ADMSCs showed a nearly 2.5 fold (P<0.05) increase when exposed to a high glucose (25 mM) medium. The percentage of insulin positive cells in the EX-4 treated group was ap- proximately 4-fold higher than in the EX-4 untreated ADMSCs.

**Conclusion:**

The present study has demonstrated that EX-4 enhances the differen- tiation of ADMSCs into IPCs. Improvement of this method may help the formation of an unlimited source of cells for transplantation.

## Introduction

Diabetes mellitus (Fig type 1) is one of the most common chronic diseases, affecting millions of people (1). Type 1 diabetes is caused by the autoimmune destruction of the insulin-secreting cells (IPCs) in the islets of the pancreas. Insulin administration does not prevent long-term complications of diabetes, because an optimal insulin dosage is difficult to maintain. Replacement of the damaged beta-cells with IPCs is considered as an ultimate cure for type 1 diabetes. The scarcity of human tissue donors has severely limited the transplantation of intact human pancreases or isolated islets (2). 

Many studies have focused on how to develop renewable sources of islet-replacement tissue. Several studies have shown that IPCs can be generated from progenitor cells of the pancreas (3), liver (4,5), pluripotent embryonic stem cells (6,7), and skin derived stem cells (8). However, the efficiency of these *in vitro* generated IPCs is low. Glucagon-like peptide 1 (GLP-1) is produced in intestinal L cells and released into the bloodstream in response to food intake. GLP-1 acts directly on beta cells, enhancing the effect of glucose in stimulating insulin secretion from these cells. When administered to diabetic mice, GLP-1 lowers blood glucose levels and stimulates insulin secretion (9). In addition, GLP-1 increases the beta-cell mass by inducing the differentiation and neogenesis of ductal progenitor cells into islet endocrine cells (10,11). It has been demonstrated that GLP-1 enhances differentiation of fetal pig progenitor epithelial cells into IPCs as well as initiating their functional maturation (12). 

GLP-1 has also been shown to stimulate proinsulin gene transcription in pancreatic betacells, slow down gastric emptying time and reduce food intake. For these reasons, GLP-1 has received much attention as a possible therapeutic agent in the treatment of obesity and type II diabetes. However, GLP-1 is rapidly degraded by dipeptidyl peptidase IV (DPP IV) (13). 

Exendin-4 (EX-4), a long-acting GLP-1 receptor agonist, is resistant to DPP IV and is now being used to replace GLP-1 in most studies. It has been reported that EX-4 has long-term beneficial effects on blood glucose levels in diabetic mice and rats (14). In humans and rats, EX-4 stimulates differentiation of pancreatic ductal cells into IPCS (15,17), and induces the expression of the GLP1 receptor in pancreatic ducts (9). EX-4 can enhance beta-cell mass by differentiation or neogenesis of precursor cells as well as increasing the replication of existing beta-cells (18,19). A previous study revealed that EX-4 increased the differentiation of bone marrow mesenchymal stem cells into IPCs (20). In the present study, we examined the possibility that EX-4 would enhance the differentiation of rat adipose-derived mesenchymal stem cells(ADMSCs) into IPCs. 

## Materials and Methods

### Cell culture

Rats were obtained from the Ahvaz Jundishapur University of Medical Sciences, Experimental Research Center, and this study was approved (cm-48) by the Ethics Committee of the same University. The rats were kept under standard laboratory conditions (12 hour-dark and 12 hour-light cycle, relative humidity 50 ± 5% and 22 ± 3˚C) for at least 1 week before the experiment and those conditions were preserved until the end of the experiment. Commercial food (pellets) and water were provided ad libitum. 

Subcutaneous adipose tissue from female Wistar rats was removed under sterile conditions, cut into small pieces and incubated to liberate the cells in 25 cm^2^ flasks containing Dulbecco’s Modified Eagle’s Medium (DMEM) and 1.0 mg/ml of collagenase. Incubations were performed for 15 minutes at 37˚C in a water bath where the flasks were shaken at a speed of 120 cycles/minutes. After 15 minutes, the flasks were vigorously mixed for 10 seconds and the contents filtered through a nylon screen (250 μm pore size) to collect any remaining non-disintegrated tissue. Thereafter the cell suspension was centrifuged at about 300 g for 3 minutes. After a homogenous cell suspension had been achieved, the cells were centrifuged at 1200 rpm for 7 minutes and the cell pellet re-suspended in 3 ml of culture medium. The cell suspension was seeded in 25 cm^2^ flasks with 5 ml DMEM and maintained at 37˚C in a humidified atmosphere with 5% CO_2_. The cultures of ADMSCs were inspected and refed every three days and passaged when the ADMSCs had reached approximately 80% confluence. As expected and previously described (21), the mesenchymal stem cells were isolated on the basis of their ability to adhere to the bottom of the flask.

The ADMSCs appeared spindle shaped in the culture. They were harvested in passage 3 and characterized as mesenchymal stem cells using flow cytometry analysis and differentiation potentials. 

### Characterization of adipose mesenchymal stem cells

Expression of cell surface markers on the ADMSCs at passage 3 prior to experiment were analyzed using flow cytometry as described previously. The cells were characterized with regard to a set of markers cluster of differentiation (CD) characteristic for ADMSCs including CD73, CD105, CD90, CD29, CD45 and CD34 (22). 

The differentiation potentials of ADMSCs were checked in specific media at passage 3. For adipocyte differentiation, cells were cultured in 1 μmol/l dexamethazone, 60 μmol/l indomethacine, 450 μl 3-isobutyl-1-methylxanthine. Adipocytes were characterized by microscopic examination and by Oil red O staining. For differentiation into osteoblasts, culture medium was supplemented with 0.1 μmol/l dexamethasone, 10 mmol/l β-glycerophosphate and 60 mmol/l ascorbate. Osteoblasts were characterized by Alizarin red staining and macroscopic examination (22). All materials and reagents were purchased from Sigma, USA. 

### Experimental design

The ADMSCs, after characterization, at passage 3, were used in the following experiment which involved one control and two experimental groups. Group 1 (control group), ADMSCs were cultured in DMEM. Group 2 and 3 ADMSCs were cultured in IPC differentiation media without EX-4 and with EX-4, respectively. For each group at least 12 flasks of culture cells were required (4 flasks for the evaluation of gene expression, 4 flasks for immunofluorescence assays, and 4 flasks for the evaluation of insulin secretion). These cultures were obtained from 7 female adult Wistar rats. 

**Fig.1:**
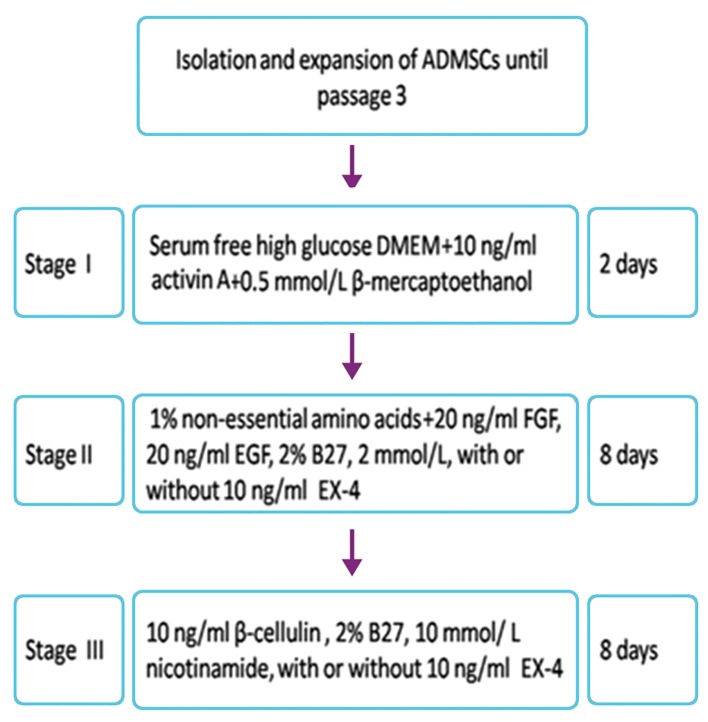
Protocol used for differentiation of ADMSCs into IPCs. ADMSCs; Adipose mesenchymal stem cells, FGF; Fibroblast growth factor, EGF; Epidermal growth factor, DMEM; Dulbecco’s Modified Eagle’s Medium and EX-4; Exendine-4.

A three-stage protocol was used to induce the IPCs. Stage 1: the cells (1×10^5^) were cultured (37˚C, 5% CO_2_) for 2 days in serum free high glucose DMEM (25 mmol/L) containing 0.5 mmol/L β-mercaptoethanol (Invitrogen, USA) and 10 ng/ml activin A (Sigma-Aldrich, USA). Stage 2: the cells then were cultured in medium containing 1% non-essential amino acids (Invitrogen), 20 ng/ml fibroblast growth factor (FGF, Sigma-Aldrich), 20 ng/ml epidermal growth factor (EGF, Sigma-Aldrich), 2% B27 (Invitrogen), 2 mmol/L L-glutamine (Hyclone Laboratories, Inc, USA) in 6-well plates for 8 days. Stage 3: the cells were cultured for an additional 8 days in new medium containing 10 ng/ml β-cellulin (Sigma-Aldrich), 2% B27, and 10 mmol/L nicotinamide (23). In the EX-4 group, 10 ng / ml EX-4 was added to the differentiation medium at stages 2 and 3. This protocol is summarized in figure 1. 

### Immunofluorescent staining

Cells harvested from the control and experimental groups were fixed with 4% paraformaldehyde (Sigma, USA) for 20 minutes and were permeabilized with 0.1% Triton X-100 (Sigma)/ phosphate buffer solution (PBS, Gibco, USA) for 10 minutes at room temperature. The cells were blocked for 30 minutes in PBS plus 0.2% Triton X-100, 1% bovine serum albumin (BSA, Sigma). Anti-insulin primary antibody (H-86: sc-9168, Santa Cruz, USA) was diluted 1: 200 in PBS and incubated overnight at 4˚C. 

The cells were rinsed three times with PBS and then incubated with fluorescently-labeled specific secondary antibody diluted in PBS with 0.5% BSA at 37˚C for 50 minutes. After washing, cells were incubated with 4´,6-diamidino2-phenylindole (DAPI, Sigma) for 10 minutes. Images were captured using an Olympus BX51 phase contrast fluorescent microscope (Olympus, Japan). From each group a minimum of four slides were examined. Immuno-staining intensity was estimated using a semi-quantitative score, the HSCORE, method. The HSCORE was calculated for each slide by application of the following algorithm (24): HSCORE=ΣPi (i+1), where i is the intensity of staining (0: no staining, 1: weak, 2: moderate, 3: strong) and Pi is the percentage of stained cells for each intensity (0 to 100%). The HSCORE of 10 random fields were evaluated for each slide and the mean HSCORE of each case was calculated. 

### Real time polymerase chain reaction (RT-PCR)

Using the RNeasy Mini kit (Qiagen, Germany), RNA was isolated from the harvested cells according to manufacturer’s instructions. cDNA was produced from the extracted RNAs using the cDNA synthesis kit based on the manufacturer’s instructions (Fermentas, Canada). 0.2 μl of each 10 pmol forward and reverse primers (Table 1), 2 μl of cDNA was added to each 25 μl of polymerase chain reaction (PCR) reaction mix, containing 12.5 μl of SYBR Green Master Mix (2x, Fermentas), and 10.1 μl DNAse free water. Information on the primers is listed in table 2. PCR amplification was done over 40 cycles using the following program: 95˚C for 10 minutes, 95˚C for 15 seconds, 5˚C for 30 seconds and 60˚C for 34 seconds. Data were analyzed using the 2^-ΔΔCT^ method (6). Gene expression in IPCs was normalized either to undifferentiated ADMSCs or adult rat islets. Expression values were corrected for the housekeeping genes β-actin and glyceraldehyde-3-phosphate dehydrogenase (*Gapdh*). The *β-actin* gene produced similar results to those with *Gapdh*.

**Table 1:** Sequence information on the primers used for real-time polymerase chain reaction (RT-PCR)


Gene name	Sequence for forward and reverse primer 5´-3´	Size(bps)	Gene Bank accession number

*Pdx-1*	F:AAACGGCACACACAAGGAGAA	150	NM-0228S2
	R:AGACCTGGCGCTTCACATG		
*Glut-2*	F:CAGCTGTCTTGTGCTCTGCTTGT	150	NM-012879
	R:GCCGTCATGCTCACATAACTCA		
*Insulin*	F:TCTTCTACACACCCATGTCCC	149	NM-019130
	R:GGTGCAGCACTGATCCAG		
*Gapdh*	F:CTCTGGTGGACCTCATGGCCTAC	105	NM-344448
	R:CAGCAACTGAGGGCCTCTCT		
*β-actin*	F:CTAAGGCCAACCGTGAAAAGA	103	NM-0311443
	R:CCAGAGGCATACAGGGACAAC		


### Radioimmunoassay (RIA)

The IPCs were incubated for 1 hour in glucose-free Krebs-Ringer bicarbonate (KRB). The cells were then incubated with KRB containing 5.56 mmol/L, 16.7 mmol/L and 25 mmol/L of glucose for 1 hour, respectively. The KRB media were collected and frozen at -80˚C until assay. Insulin assay was performed by RIA using a commercially available rat RIA kit (Millipore, Germany) according to the manufacturer’s instructions. Determinations were carried out in quadruplicate and the means and standard deviations were obtained. 

### Statistical analysis

Comparisons of multiple (>3) group means were performed using one-way ANOVA and post hoc procedures based on Newman-Keuls tests. The Kruskal-Wallis and Mann-Whitney tests were used for comparisons of semi-quantitative immunostaining. A P<0.05 was considered statistically significant. 

## Results

### Characterization of adipose mesenchymal stem cells

**Fig.2:**
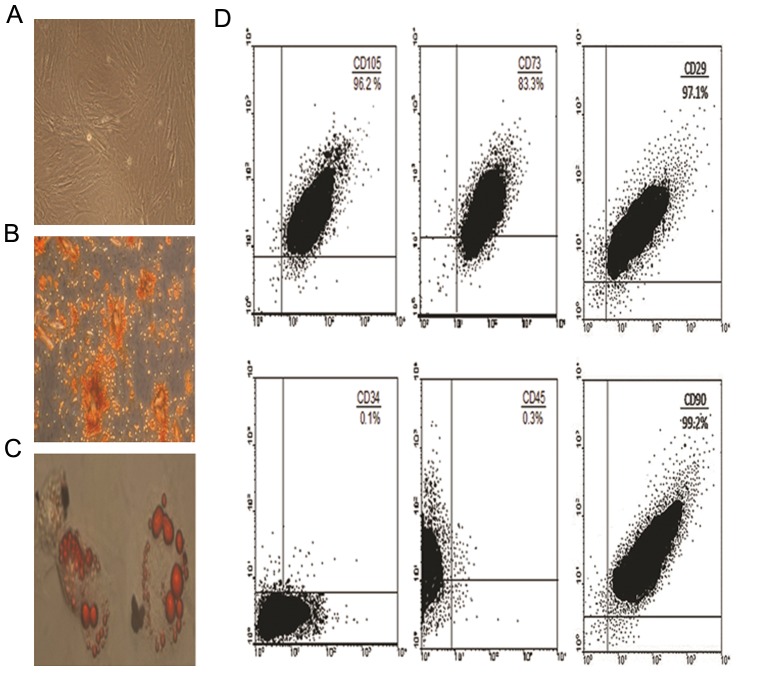
Characteristics of ASCs. A. ASCs at passage 3. The cells have a fibroblast-like morphology, B. Potential differentiation of ASCs into osteogenic cells (Alizarin red staining), C. Potential differentiation of ASCs into adipogenic cells (Oil red O staining) and D. Flow cytometric analysis of surface-marker expression on ASCs (magnifications: A; ×100, B; ×250 and C; ×400). ASCs; Adipo srem cells and CD; Cluster of differentiation.

ADMSCs were typically adherent, spindleshaped and fibroblast-like at passage 3. They were characterized as mesenchymal stem cells using flow cytometry analysis and differentiation potentials at passage 3. Cell surface markers detected by flow cytometry revealed that ADMSCs expressed high levels of CD90 (99.2%), CD29 (97.1%), CD105 (96%) and CD73 (83%), whereas expression of CD34 and CD45 was very low. ADMSCs in the presence of appropriate media were capable of *in vitro* differentiation into osteoblasts and adipocytes. These results are shown in figure 2. 

### Morphological changes during differentiation of adipose-derived mesenchymal stem cells 

**Fig.3:**
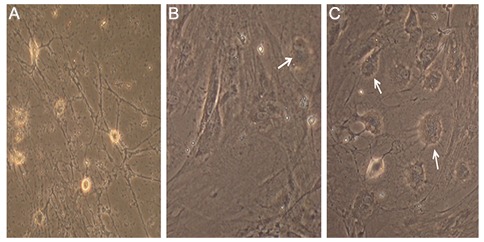
Morphological changes of ADMSCs. A. ADMSCs in DMEM, B. ADMSCs in IPC differentiation media without EX-4 and C. ADMSCs in IPC differentiation media with EX-4. Arrows indicate IPCs (magnifications: A; ×250, B and C; ×400). ADMSCs; Adipose mesenchymal stem cells, DMEM; Dulbecco’s Modified Eagle’s Medium, EX-4; Exendine-4 and IPCs; Insulin-producing cells.

Under an inverted microscope, ADMSCs were typically adherent, spindle-shaped and fibrocyte-like at passage 3 (Fig .1). The ADMSCs cultured in non-differentiation inducing media (control group) showed various shapes including spherical, neuron-like cells or glial-like cells (Fig .2A). In the presence of differentiation media with EX-4, the ADMSCs displayed a spherical morphology with confluence similar to pancreatic islet-like clusters. A spherical morphology seemed to be less common in cells cultured in differentiation media without EX-4 than those exposed to differentiation media containing EX-4 (Fig .3A,B). 

**Fig.4:**
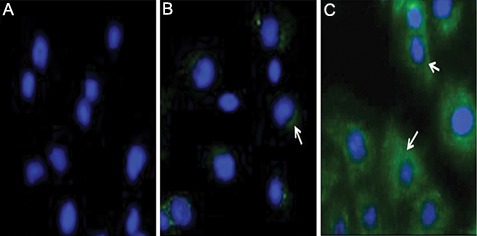
Immunofluorescence analysis of insulin. A. ADMSCs in DMEM, B. ADMSCs in IPC differentiation media without EX-4 and C. ADMSCs in IPC differentiation media with EX-4 (magnifications: ×250). ADMSCs; Adipose mesenchymal stem cells, DMEM; Dulbecco’s Modified Eagle’s Medium, EX-4; Exendine-4 and IPCs; Insulin-producing cells.

### Immunofluorescence staining

**Fig.5:**
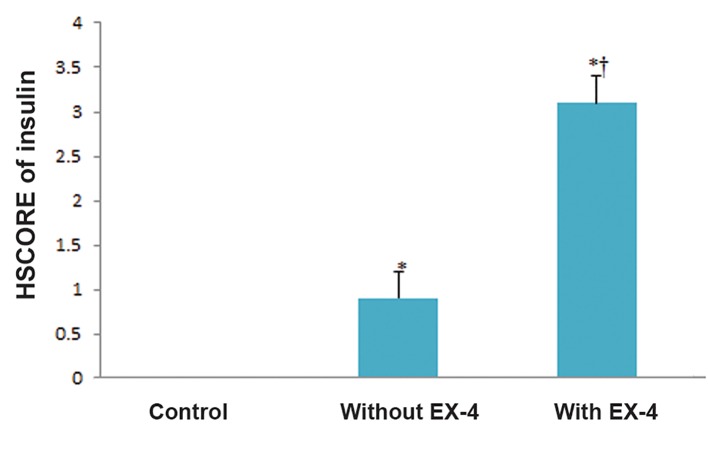
HSCORE assessments of insulin immune-staining. Values are expressed as mean ± SD. *, †; Symbols respectively indicate comparison to control and EX-4 untreated IPCs (P<0.001), EX-4; Exendine-4 and IPCs; Insulin-producing cells.

Expression of insulin proteins showed up as green in the immunofluorescence assay (Fig .3). In EX-4-untreated ADMSC-derived IPCs there was a marked increase in the percentage of IPCs expressing insulin-approximately 39% of examined cells-of which 35% showed weak, 4% moderate and 0% strong cytoplasmic staining for insulin. In EX-4-treated ADMSC-derived IPCs, the percentage of insulin expressing cells was significantly increased to 87%, of which 7% showed weak, 20% moderate and 60% strong cytoplasmic staining for insulin. Overall, HSCORE assessments showed that insulin expression in 3 dimensional (3D) culture cells was about 2.5 fold higher than in the 2D culture cells. These results are shown in figures 4 and 5.

### Gene expression of adipose mesenchymal stem cells-derived insulin-producing cells

**Fig.6:**
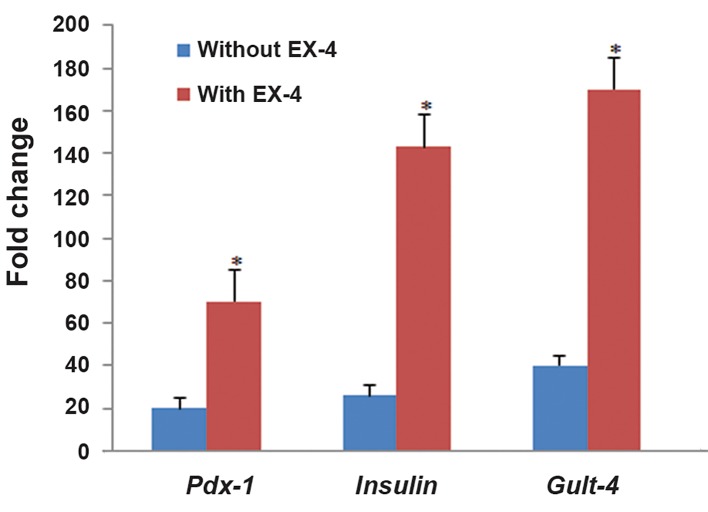
Fold change gene expression in various groups. Values are expressed as mean ± SD. Star symbol indicates comparison to control and EX-4 untreated IPCs. *; P<0.001, EX-4; Exendine-4 and IPCs; Insulin-producing cells.

To determine whether the ADMSCs had undergone pancreatic differentiation, gene expression profiles for pancreatic cell differentiation markers were assessed using real time PCR (RTPCR). As illustrated in figure 6 and Table 2, low expression of *Pdx-1, Glut-2* and *Insulin* was detected in undifferentiated ADMSCs (control). Compared to EX-4-untreated ADMSC-derived IPCs, expression of *Pdx-1, Glut-2* and *Insulin* genes in EX-4-treated ADMSC-derived IPCs showed nearly 3.5 fold, 4.3 fold and 5.7 fold (P<0.001) increases respectively. 

**Table 2:** Ct mean and ∆Ct of control and experimental group


	Ct mean	ΔCt


**Control**		
*Pdx-1*	21.8	2.1
*Insulin*	20.9	1.2
*Glut-2*	21.1	1.4
*Gapdh*	19.7	

**Without EX-4**		
*Pdx-1*	33.9	6.5
*Insulin*	33.4	5.6
*Glut-2*	34.1	6.7
*Gapdh*	27.4	

**With EX-4**		
*Pdx-1*	36.9	8.3
*Insulin*	37.0	8.5
*Glut-2*	37.4	8.8
*Gapdh*	28.4	


Ct; Threshold cycle and Δct; The difference in threshold cycles for the target and control samples.

### Insulin release in response to glucose stimulation

**Fig.7:**
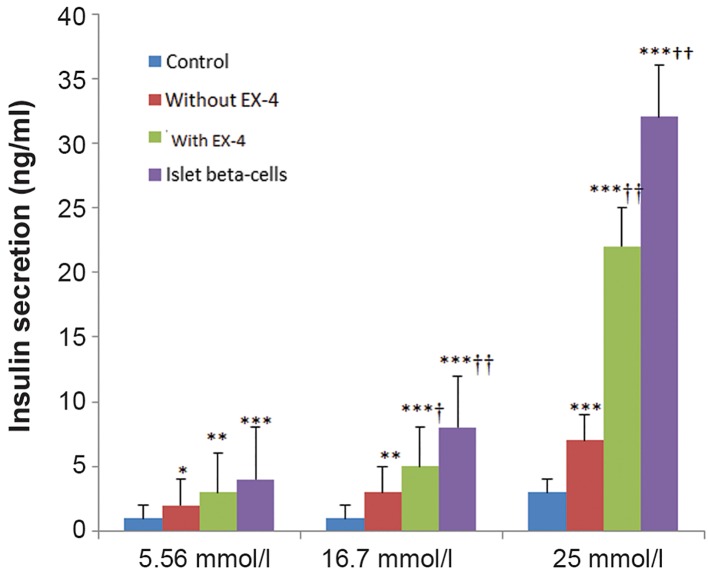
Insulin excretion changes in various groups. Values are expressed as mean ± SD. *, †; Symbols respectively indicate comparison to control and EX-4 untreated IPCs, *; P<0.05, **; P<0.01, ***; P<0.001, †; P<0. 01, ††; P<0.001, EX-4; Exendine-4 and IPCs; Insulin-producing cells.

The insulin concentration in each group was compared with a one way ANOVA and was followed by pair-wise comparisons. Adult rat beta cells, used as positive controls (the kind gift of Dr. A. Ahangarpour, Cell and Molecular Research Center, Iran) showed high levels of insulin in the presence or absence of a glucose challenge. The cultured ADMSCs in the control group showed very low levels of insulin in the presence or absence of a glucose challenge. The EX-4-untreated ADMSC-derived IPCs could release insulin at a low concentration of glucose (5.56 mmol/L) and release approximately 20 fold insulin under a glucose challenge (25 mmol/L) (P<0.01). Insulin secretion was significantly increased in EX-4-treated ADMSCderived IPCs at a low concentration of glucose (2 fold) and under a glucose challenge, compared to the EX-4-untreated ADMSC-derived IPCs (P<0.01). Insulin secretion of the EX4-treated ADMSC-derived IPCs was significantly decreased at a glucose concentration of 16.7 mmol/L and under a glucose challenge (1.4 fold) compared to the islet beta-cells (P<0.01, Fig .7). 

## Discussion

The results of this study have demonstrated that EX-4 can effectively enhance differentiation of ADMSCs into IPCs. In this study the existence of IPCs was confirmed by morphological analysis, analysis of the expression pattern of islet-specific genes, and insulin synthesis and secretion. The induced IPCs were morphologically similar to pancreatic islet-like cells (23). More interestingly, they not only produced insulin but could also secret insulin in response to stimulation with different concentrations of glucose. These effects were stronger in the presence of EX-4. 

The expression of *Pdx-1* in EX-4-treated ADMSC-derived IPCs was markedly increased in comparison to EX-4-untreated ADMSC-derived IPCs. PDX is a pancreatic homeoprotein that is critical for the development of both the endocrine and exocrine pancreas. *Pdx-1* mediates glucose-responsive stimulation of insulin gene transcription (25). Its capacity to activate gene transcription in a tissue specific mode is dependent on its ability to interact with other transcription factors (26). Pdx-1 activates the promoters of several genes involved in the maturation of beta cells, including *Insulin*, glucose transporter 2 (*Glut-2*), glucokinase, and islet amyloid polypeptide (27). Movassat et al. (19) have reported that EX-4 up-regulates expression of *Pdx-1* and enhances differentiation and maturation of human fetal pancreatic cells. Aviv et al. (28) showed that EX-4 stimulated liver cell proliferation and enhanced the Pdx-1-induced liver to pancreas differentiation process. 

High expression of *Insulin 2* and *Glut-2* genes in EX-4-treated ADMSC-derived IPC was also observed in the present study. It is well known that expression of these genes confirms the differentiation and full functionality of IPCs. In pancreatic beta-cells, glucose uptake is controlled by Glut-2, which is essential in the mechanism of glucoseinduced insulin secretion (29). 

Li et al. (30) have reported that EX-4 increased insulin secretion in differentiated beta cells from mouse embryonic stem cells. Additionally, RIA analysis demonstrated a significant increase in insulin secretion upon a glucose challenge in EX4-treated ADMSC-derived IPCs compared to EX4-untreated ADMSC-derived IPCs. As shown in our results, the percentage of IPCs was markedly increased in the presence of EX-4. These findings indicate that more mature Insulin-Secreting Cells can be generated in presence of EX-4. Park et al. (31) have demonstrated that EX-4 and exercise promotes beta-cell function and mass in the islets of diabetic rats. Stoffers et al. (32) have shown that exposure to EX-4 in the postnatal period reverses the adverse consequences of fetal programming and prevents the development of diabetes in adulthood. It has been revealed that GLP-1 promotes the expansion of pancreatic beta-cell mass by stimulating neogenesis as well as the proliferation of existing beta-cells (33,34). Administration of EX-4 during regeneration after 90% partial pancreatectomy in rat results in a sustained improvement in glucose homeostasis associated with a 40% increase in beta-cell mass due to increases in both neogenesis and replication (9). 

## Conclusion

This study has demonstrated that EX-4 can enhance the differentiation of ADMSCs into IPCs. The exact mechanism by which EX-4 enhances this differentiation was not examined in this study. Further experiments are needed to clarify the mechanisms by which EX-4 affects mesenchymal stem cell differentiation. 
